# Using the theory of planned behavior model to predict factors influencing breastfeeding behavior among preterm mothers at week 6 postpartum: the mediating effect of breastfeeding intention

**DOI:** 10.3389/fpsyg.2023.1228769

**Published:** 2023-09-08

**Authors:** Rong Huang, Hui Han, Lijing Ding, Yi Zhou, Yanwen Hou, Xiao Yao, Chenting Cai, Xiaohan Li, Jianqi Song, Shuying Zhang, Hui Jiang

**Affiliations:** ^1^Shanghai First Maternity and Infant Hospital, School of Medicine, Tongji University, Shanghai, China; ^2^School of Medicine, Tongji University, Shanghai, China

**Keywords:** breastfeeding behavior, intention, mediating effect, preterm, theory of planned behavior

## Abstract

**Background:**

Exclusive breastfeeding (EBF) in the first 6 weeks postpartum is key to continued breastfeeding. This study aimed to explore the role of EBF-related predictors (particularly breastfeeding intention) in breastfeeding behavior among preterm mothers at week 6 postpartum based on the theory of planned behavior (TPB).

**Methods:**

A total of 352 mothers of preterm infants were recruited, 340 of whom participated in this study. Prior to discharge, participants completed the Chinese versions of the modified Breastfeeding Attrition Predictive Tool, the Breastfeeding Knowledge Questionnaire (BKQ), the Infant Feeding Intention, and the Edinburgh Postnatal Depression Scale. Responses to the items of the Breastfeeding Behavioral Questionnaire (BBQ) were also collected by telephone at week 6 postpartum. The final analyses included 321 participants who completed the full two-wave data collection.

**Results:**

The fitness indices of the modified TPB model were acceptable. Breastfeeding knowledge and EBF before discharge positively impacted breastfeeding intention, whereas depression had a negative impact. Before discharge, breastfeeding intention fully mediated the impacts of breastfeeding attitude, social and professional support, knowledge, depression, and EBF on breastfeeding behavior and partially mediated the influence of perceived breastfeeding control on breastfeeding behavior.

**Conclusion:**

These findings indicate that TPB accurately predicts breastfeeding behavior among preterm mothers at week 6 postpartum, and breastfeeding intention is key to the above-mentioned EBF-related factors and breastfeeding behavior. The findings underline the need for further longitudinal studies and corresponding interventions for preterm mothers with a high risk of EBF attrition.

## Introduction

Breastfeeding plays a significant role in reducing morbidity and mortality (Cleminson et al., 2016) and promoting long-term health for preterm infants (gestational age < 37 weeks; Størdal et al., 2017). Individuals who were not exclusively breastfed as infants have higher rates of health problems as they age, including respiratory infections, allergies, digestive problems, malnutrition, diabetes, obesity (Victora et al., 2016), and childhood and adolescent cancers (Rafizadeh et al., 2019). Breastfeeding has also been associated with the intelligence and academic performance of children (Belfort et al., 2016). The World Health Organization (WHO) recommends exclusive breastfeeding (EBF) for all infants (including preterm infants) because of the significant health benefits to mother–infant dyads (World Health Organization, 2002; Britton et al., 2006). According to WHO, EBF refers to “giving no other food or drink—not even water—except breast milk. It, however, allows the infant to receive oral rehydration salts (ORS), drops, and syrups (vitamins, minerals, and medicines)” (World Health Organization, 2017). However, the rate of EBF in China is currently unsatisfactory, especially in the neonatal intensive care unit (NICU) setting, where fewer than 15% of mothers practice EBF (Shi and Zhang, 2015). Research also found that breastfeeding attrition occurs in 32 to 58% of breastfeeding mothers within the first 6 weeks (Janke, 1992), indicating that EBF in the first 6 weeks postpartum is key to breastfeeding continuation.

Multiple factors contribute to breastfeeding as a social behavior (Guo et al., 2016). Clarifying the factors associated with EBF in mothers of preterm infants is critical to helping educate women on the importance of EBF (World Health Organization, 2002). Factors related to breastfeeding can be generally categorized into demographics (such as age, education, and parity) and psychosocial characteristics of preterm mothers (including previous breastfeeding experience, mother–infant separation, breastfeeding intention, maternal leave time, breastfeeding attitude, breastfeeding self-efficacy, breastfeeding knowledge and skill, family and social support, nipple problems, insufficient milk, EBF before discharge, and depression [per the Edinburgh Postnatal Depression Scale score]; Blyth et al., 2004; Quan and Yang, 2005; Wilhelm et al., 2008; Gilmour et al., 2009; Whalen and Cramton, 2010; Demirci et al., 2013; Manno et al., 2015; Wang et al., 2019). Characteristics of preterm infants also contribute to breastfeeding success and include factors such as gestational age, birth weight, dysphagia, insufficient oral sucking power, and time to start sucking breast milk after delivery (Lou et al., 2014; Sonko and Worku, 2015; Tao et al., 2016; Geddes et al., 2018; Wang et al., 2019). Encouragingly, some of these factors can be improved through intervention (Fishbein and Ajzen, 2010; Insaf et al., 2011; Saffari et al., 2016; Thomas-Jackson et al., 2016; Araban et al., 2018; Suárez-Cotelo et al., 2019), including mothers’ breastfeeding intention, attitude, subjective norms, knowledge, depression, perceived level of control over breastfeeding (i.e., perceived behavioral control), and EBF before discharge (Fishbein and Ajzen, 2010; Insaf et al., 2011; Saffari et al., 2016; Thomas-Jackson et al., 2016; Araban et al., 2018; Suárez-Cotelo et al., 2019).

Breastfeeding intention, attitude, perceived behavioral control, and subjective norm are all considered key factors of breastfeeding initiation and duration (Janke, 1992; Bai et al., 2010; Tengku Ismail et al., 2016). Furthermore, interventional studies suggest that breastfeeding knowledge, depression, and EBF before discharge influence breastfeeding intention (Insaf et al., 2011; Thomas-Jackson et al., 2016; Suárez-Cotelo et al., 2019), although the relationships between each of these factors remain unclear, particularly those surrounding normative beliefs (Göksen, 2002; Kloeblen-Tarver et al., 2002) and perceived behavioral control (Avery et al., 1998; Bai et al., 2010). Therefore, identifying the factors that are significantly associated with breastfeeding intention and behavior and analyzing the interactions between these factors are essential to the development of a tailored breastfeeding promotion intervention for preterm mothers.

**Figure 1:**
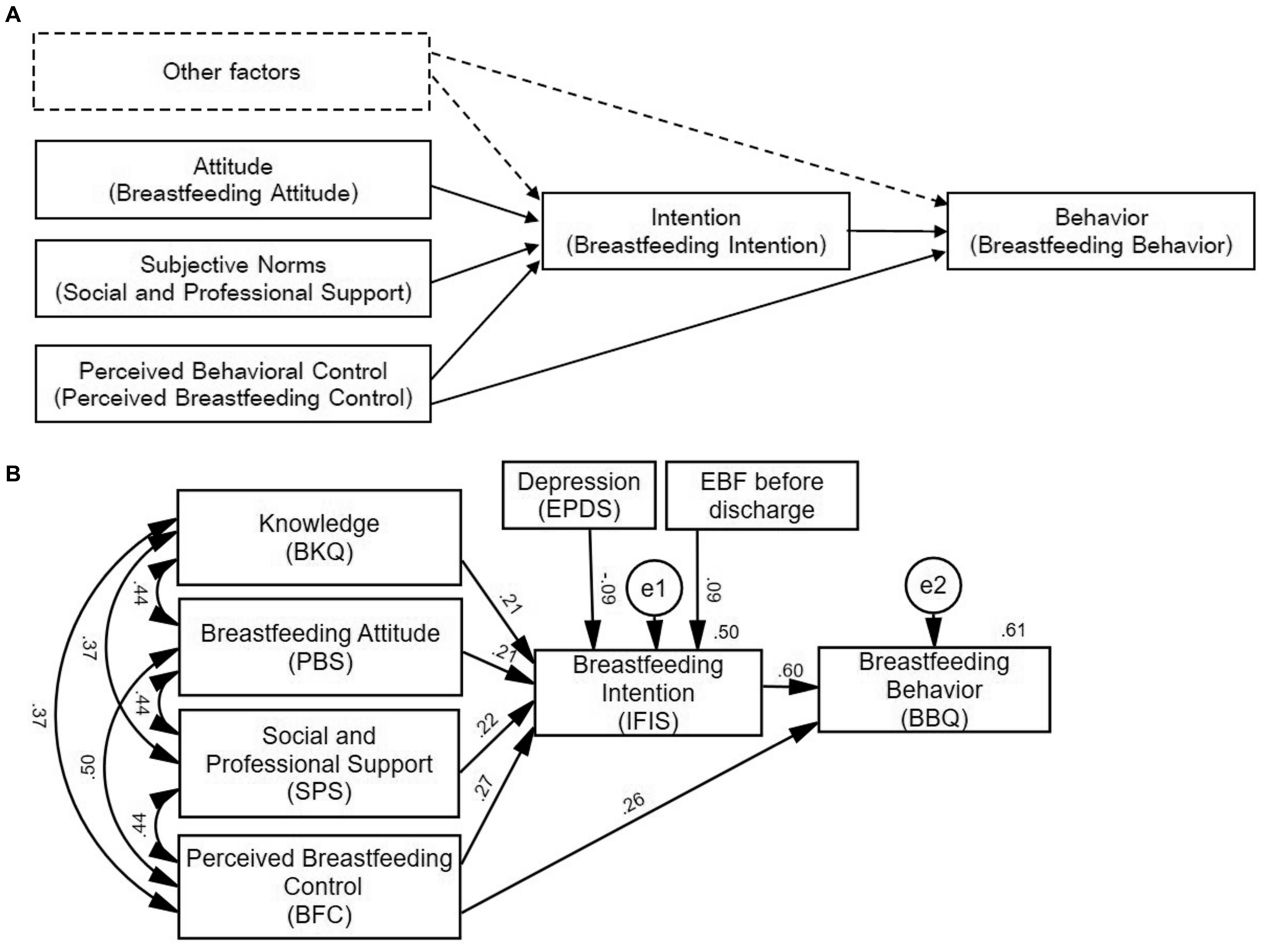
**(A)** Hypothesized model of breastfeeding behavior of preterm mothers based on the theory of planned behavior. The variables in the solid box represent the application of the framework of the theory of planned behavior in this study, while the variables in the dashed box represent the new variables introduced in this study. Other factors include the socio-demographics of the dyads and the preterm mothers’ knowledge, EBF before discharge, and depression. **(B)** Modified model of breastfeeding behavior of preterm mothers based on the theory of planned behavior. PBS, SPS, and BFC were the total scores of three subscales of the Chinese version of the Modified Breastfeeding Attrition Predictive Tool (Positive Breastfeeding Sentiment Attitudinal Scale, Social and Professional Support Scale, and Breastfeeding Control Scale, respectively); and BKQ, IFIS, BBQ, and EPDS were the total scores of the Chinese versions of the Breastfeeding Knowledge Questionnaire, the Infant Feeding Intention Scale, the Breastfeeding Behavioral Questionnaire, and the Edinburgh Postnatal Depression Scale, respectively.

Promoting breastfeeding can be strengthened by incorporating health-based behavior theories including the theory of reasoned action (TRA; Ajzen and Madden, 1986), the theory of planned behavior (TPB), and the knowledge, attitude, and practice (KAP) model (Gebeyehu et al., 2023). Although the TRA does not account for personal control factors in the decisions of pregnant women about breastfeeding, a modified version, the TPB (Fishbein and Ajzen, 2010), includes perceived behavioral control (PBC). This addition significantly improved the explanatory and predictive power of behavior (Wang and Jiang, 2021) compared with the KAP. The TPB also considers the effects of social, attitudinal, and behavioral determinants (Esquerra-Zwiers et al., 2022) and is an ideal theoretical framework to understand human behavior (Ajzen, 1991). Briefly, the TPB framework suggests that the intention to perform a behavior is linked to the actual behavioral performance. This framework has been widely used to better understand parent behaviors, including breastfeeding in full-term infants (Khoury et al., 2005; McMillan et al., 2009; Guo et al., 2016). Indeed, a recent study reported that PBC explains 65% of the EBF intention of mothers and predicts 79% of the variance in EBF (Bajoulvand et al., 2019). A previous study explored the factors related to EBF attrition of preterm mothers at week 6 postpartum through binary logistic regression analysis (Huang et al., 2023) and found that breastfeeding attitude, social and professional support, perceived control, and knowledge were associated with breastfeeding attrition. Thus, the purpose of this study was to explore the role of EBF-related predictors (particularly breastfeeding intention) in breastfeeding behavior among preterm mothers at week 6 postpartum based on the TPB. The Infant Feeding Intention Scale (Nommsen-Rivers and Dewey, 2009) and the Breastfeeding Behavioral Questionnaire (Alami et al., 2014; Bajoulvand et al., 2019) were used to assess breastfeeding intention and behavior. Structural equation modeling (SEM) was used to examine the following hypotheses (Figure 1A) via exploration of the paths between the factors (namely breastfeeding attitude, subjective norms, perceived behavioral control, intention, knowledge, EBF before discharge, and depression) related to breastfeeding behavior at week 6 postpartum among mothers of preterm infants.

*H1*: Breastfeeding attitude, social and professional support (subjective norms), perceived breastfeeding control, social demographics, breastfeeding-related knowledge, EBF before discharge, and depression are directly associated with breastfeeding intention among preterm mothers.

*H2*: Breastfeeding intention (and its antecedents) is directly associated with breastfeeding behavior among preterm mothers.

*H3*: Breastfeeding intention mediates the association between seven antecedents of intention and breastfeeding behavior among preterm mothers.

## Methods

### Study design, setting, and ethical approval

A prospective observational study was designed and conducted from October 2021 to July 2022 in the obstetric wards of Shanghai First Maternity and Infant Hospital in Shanghai, China. This tertiary specialized hospital houses 400 obstetric beds, 14 labor beds, and 110 beds for the neonatal ward; its annual delivery volume is approximately 25,000–30,000 infants. Preterm infants whose gestational age is <36 weeks are routinely admitted to the NICU. Furthermore, those who are born between 36 weeks and 37 weeks of gestational age are routinely admitted to the NICU for a 6-h observation and returned to their mothers in the obstetric ward if no special condition is detected during their NICU stay. Following vital sign stabilization, fresh breast milk is the preferred choice of enteral nutrition during the stay of a premature infant in the NICU, followed by breast milk refrigerated for less than 24 h. Family members may apply for donated milk due to special conditions of preterm mothers, such as illness, medication-induced prohibition of breastfeeding, lack of milk in the first 3 days, or if their preterm infant has a gestational age ≤34 weeks and/or weight <1,800 g. If the mother has no or insufficient milk, she can also choose preterm infant formula. For preterm infants with clinical gestational age ≤34 weeks, weight <1,800 g, or high-risk factors for malnutrition, human milk fortifiers can be added to breast milk or donated milk to meet nutritional needs. The study was approved by the Ethics Committee of Shanghai First Maternity and Infant Hospital (approval number KS21355).

### Sampling

A convenience sampling strategy was used, and eligible participants were recruited from the obstetric ward of the designated hospital. Inclusion criteria of the mothers were (1) aged >20 years old, (2) gestational age ≥28 weeks and <37 weeks, (3) singleton pregnancy, and (4) provided informed consent. Mothers were excluded from the study if they (1) took drugs that may affect breast milk secretion during pregnancy and postpartum, (2) had a diagnosed intellectual disability or endocrine system disease, (3) had at least one type of communication disorder, (4) had a preterm infant with congenital malformations, or (5) had a preterm infant that could not be breastfed due to disease or other reasons.

While various recommendations exist for determining sample size in SEM studies (Gefen et al., 2000; Boomsma and Hoogland, 2001; Hoe, 2008; Iacobucci, 2010), we determined 24 free parameters in this study and a minimum sample size of 120 mother–infant dyads. After taking the probability of outlier data into account, 6 times the number of free parameters was estimated (144 samples) for a calculated sample size of 216. Considering a probable data loss of 30%, we determined the final sample size should match or exceed 281 participants.

### Data collection

The researchers (XY, LJD, CTC, and JQS) introduced the purpose of the study to eligible mothers prior to discharge. Interested mothers then provided their contact details to the researchers, and written informed consent was obtained from each participant prior to data collection. Participants completed an initial set of questionnaires, and at week 6 postpartum, researchers followed up with participants to gauge their responses to the Breastfeeding Behavioral Questionnaire.

### Measurements

Demographic information of the participants and their preterm infants included (a) maternal information (age at delivery, education level, monthly income, gestational age, gravidity, parity, type of delivery, postpartum depression, EBF before discharge, nipple depression, and history of breastfeeding) and (b) demographic information of preterm infants (sex, birth weight, and time to start suckling breast milk after delivery).

Five surveys were used in the current study following methods previously reported: the Chinese version of the Modified Breastfeeding Attrition Predictive Tool (modified BAPT; Janke, 1994; Dick et al., 2002; Cai et al., 2023), the Breastfeeding Knowledge Questionnaire (BKQ; Ouyang et al., 2012, 2016), the Edinburgh Postnatal Depression Scale (EPDS; Cox et al., 1987; Gibson et al., 2009; Lee et al., 2019; Liu et al., 2020), the Infant Feeding Intention Scale (IFIS; Nommsen-Rivers and Dewey, 2009), and the Breastfeeding Behavioral Questionnaire (BBQ; Alami et al., 2014; Bajoulvand et al., 2019).

Three subscales using 5-point Likert scales from the Chinese version of the modified Breastfeeding Attrition Predictive Tool (BAPT) were utilized—the Positive Breastfeeding Sentiment Attitudinal Scale (PBS, 12 items), the Social and Professional Support Scale (SPS, 11 items), and the Breastfeeding Control Scale (BFC, 10 items)—to measure breastfeeding attitude (ATT), social and professional support (SPS), and perceived breastfeeding control (PBC), respectively. Cronbach’s α coefficients of the PBS, SPS, and BFC scales were 0.878, 0.931, and 0.921, respectively (Cai et al., 2023), and the reliabilities of a 2-week retest were 0.765, 0.778, and 0.530, respectively (Cai et al., 2023). The item-level content validity index (CVI) was 0.80–1.00 (Cai et al., 2023).

The Chinese version of the BKQ (Ouyang et al., 2012, 2016) includes 18 questions evaluating knowledge and awareness of the benefits of breastfeeding and the management of common lactation issues. Cronbach’s α was 0.82, and the CVI was 0.87 (Ouyang et al., 2012).

The EPDS (Cox et al., 1987) includes 10 items using 4-point response options to capture symptoms of depression. Cronbach’s α for the Chinese version of EPDS was 0.862 (Liu et al., 2020), and the CVI was 0.93 (Lee et al., 2019). A cutoff score of ≥13 was used in this study to indicate major or probable depression (Gibson et al., 2009).

The IFIS (Nommsen-Rivers and Dewey, 2009) quantitatively measures maternal breastfeeding intentions. The scale includes five items using 5-point response options ranging from 0 to 4, although only three of these items relevant to the purpose of the study were used: “I am planning to only formula feed my baby (I will not breastfeed at all).” “I am planning to at least give breastfeeding a try.” “When my baby is 6 weeks postpartum, I will be breastfeeding without using any formula or other milk.” Cronbach’s α for the IFI was 0.90, and the CVI of items 1 to 3 was 0.70, 0.76, and 0.67, respectively (Nommsen-Rivers and Dewey, 2009).

Finally, the BBQ (Alami et al., 2014) utilizes TPB to evaluate actual breastfeeding behavior. The questionnaire consists of four items, each of which has a score ranging from 1 to 5 points. The total score ranges from 5 to 20, with higher scores indicating more stable exclusive breastfeeding behavior. Cronbach’s α for the BBQ was 0.79, the intra-group correlation coefficient (ICC) was 0.81, and the CVI was 0.65 to 0.99 (Bajoulvand et al., 2019).

### Statistical analysis

SPSS statistics version 21.0 software (Statistics 21, SPSS, IBM, United States) was used for the preliminary analysis. The relationships between mean scores of breastfeeding intention and behavior with dichotomous and polychotomous demographic variables were tested by the independent-sample *t*-test and variance analysis, respectively, and Cohen’s *d* and *η*_p_^2^ were used for effect size estimates, respectively (Cohen, 1988). The relationship between continuous variables of breastfeeding behavior and PBC, ATT, SPS, knowledge, and intention were tested using Pearson correlation analyses, and the *r*-value was used for effect size estimate (Cohen, 1988).

The study was based on the basic framework of the TPB and included additional factors such as socio-demographics of the dyads, preterm mothers’ breastfeeding knowledge, depression, and EBF before discharge (Quan and Yang, 2005; Zhang, 2014; Suárez-Cotelo et al., 2019) for the development of the hypothesized model. SEM using maximum-likelihood estimations was performed through AMOS 26.0 (IBM SPSS Amos 26 Graphics) to examine this model, and the fitness indices were confirmed using the *χ*^2^/df, the root mean square error of approximation (RMSEA), the goodness-of-fit index (GFI), the adjusted goodness-of-fit index (AGFI), the Tucker–Lewis coefficient (TLI), the comparative fit index (CFI), the relative fit index (RFI), the normed fit index (NFI), and the incremental fit index (IFI). Fit was considered acceptable if the following cutoffs were met: *χ*^2^/df < 3.0; RMSEA <0.08; and TLI, CFI, RFI, NFI, and IFI > 0.90 (Hu and Bentler, 1999; Byrne, 2010; Jansen et al., 2016; Hwang and Sim, 2021). The mediating effect of breastfeeding intention was evaluated using 2000 bootstrapping samples and 95% bias-corrected confidence intervals (CIs; Lei et al., 2022). When the CI excluded zero, the effect was considered significant. When the total effect of a variable was significant, if both the indirect and direct effects were significant, the variable had a partial mediating effect. Conversely, if the direct effect was not significant and the indirect effect was significant, the variable had a full mediating effect (Hayes and Scharkow, 2013). The significance level of all variables was set to α = 0.05.

## Results

### Demographic characteristics and evaluations of breastfeeding intention and behavior

**Figure 2:**
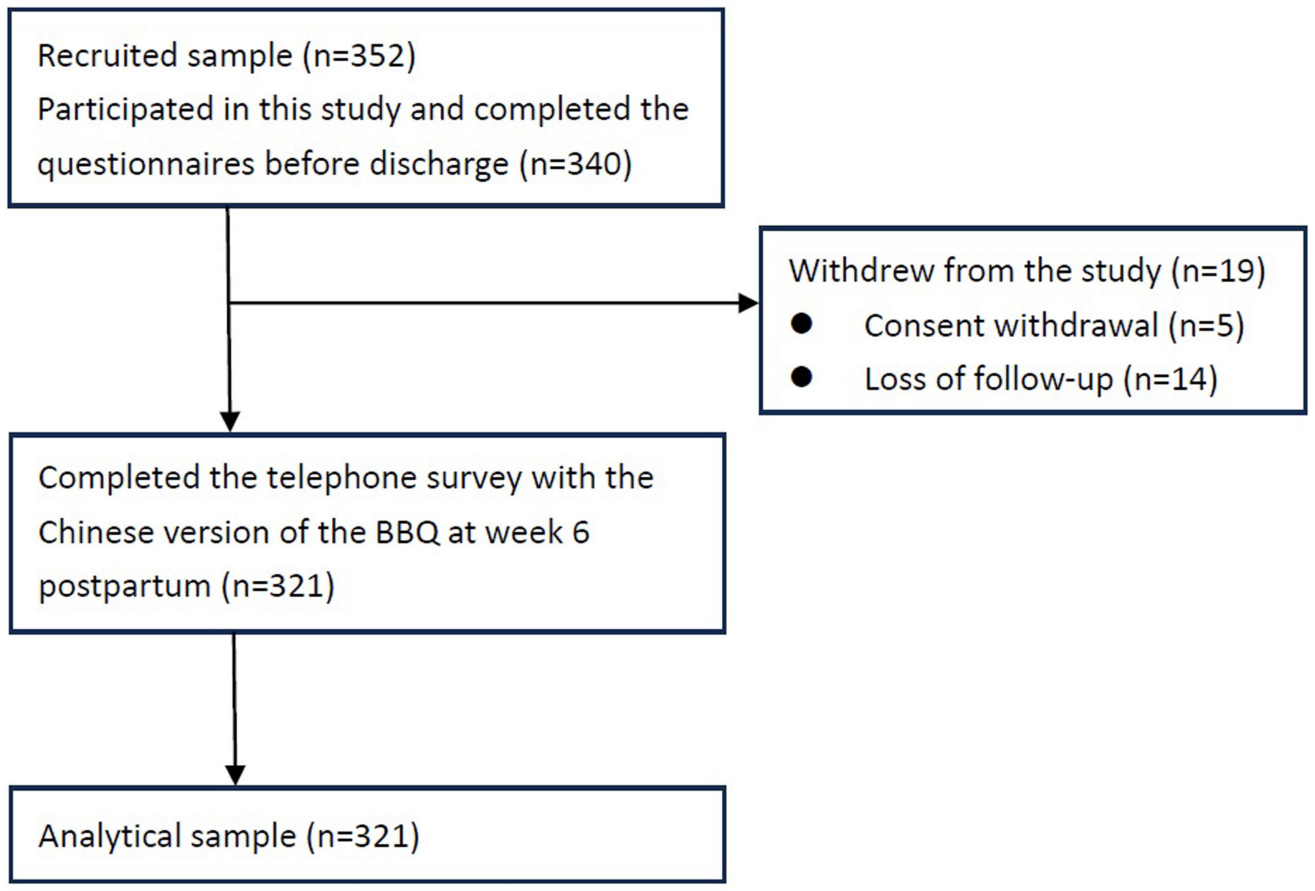
Flow diagram of the study process.

A total of 352 mothers of preterm infants were recruited, 340 of whom participated in this study and completed the questionnaires before discharge. A total of 321 participants completed the telephone survey with the Chinese version of the BBQ at week 6 postpartum; 19 participants withdrew from the study due to withdrawal of consent (*n* = 5) and loss of follow-up (*n* = 14; Figure 2).

Table 1 outlines the demographics, psychosocial characteristics, and evaluations (including EBF before discharge, nipple depression, and total score of EPDS) of the dyads (*n* = 321), as well as the relationships between demographic and psychosocial data and evaluations with breastfeeding intention and behavior of the participants. The participants with higher EPDS scores (total score ≥ 13) were less likely to have breastfeeding intentions (Cohen’s *d*: −1.009) and behavior (Cohen’s *d*: −0.732) at week 6 postpartum (*p* < 0.01), with large and medium effects, respectively. The participants with EBF before discharge had higher breastfeeding intention (Cohen’s *d*: 0.752) and behavior (Cohen’s *d*: 0.715) at week 6 postpartum (*p* < 0.01; Table 1). No significant relationships between other socio-demographic factors were found with breastfeeding intention and behavior (*p* > 0.05).

**Table 1:** Socio-demographics and the relationships between preterm infants and their mothers at week 6 postpartum with breastfeeding intention and behavior.

Characteristics	*N* (%; *n* = 321)	Breastfeeding intention score^a^ (^−^x ± s)	*t*/*F*	Cohen’s *d/η*_p_^2^	Breastfeeding behavior score^b^ (^−^x ± s)	*t*/*F*	Cohen’s *d/η*_p_^2^
**Age at delivery (years)**							
<35	246 (76.6)	6.193 ± 1.282	0.559	0.074	15.95 ± 2.686	1.153	0.152
≥35	75 (23.4)	6.100 ± 1.219			15.55 ± 2.575		
**Education level**							
Higher education	40(12.5)	5.775 ± 1.160	2.288	0.014	15.73 ± 2.418	0.057	0.000
Bachelor degree	210 (65.4)	6.239 ± 1.279			15.87 ± 2.703		
Master degree	71 (22.1)	6.197 ± 1.263			15.89 ± 2.702		
**Monthly income (RMB)**							
≤5000元	11 (3.4)	6.455 ± 1.214	0.398	0.004	15.82 ± 2.926	0.823	0.003
5,001–10000元	72 (22.4)	6.078 ± 1.261			15.83 ± 2.512		
10,001–15000元	76(23.7)	6.243 ± 1.210			16.11 ± 2.543		
>15000元	162 (50.5)	6.160 ± 1.304			15.75 ± 2.778		
**Gestational age (weeks)**							
28–31^+6^	26 (8.1)	6.173 ± 1.216	0.006	0.001	16.31 ± 2.558	0.901	0.187
32–36^+6^	295 (91.9)	6.172 ± 1.273			15.82 ± 2.671		
**Gravidity**							
1 time	161 (50.2)	6.153 ± 1.322	0.057	0.000	15.97 ± 2.656	0.420	0.003
2 times	77 (24.0)	6.169 ± 1.266			15.86 ± 2.822		
3 times or above	83 (25.8)	6.211 ± 1.166			15.64 ± 2.535		
**Parity**							
Primipara	241 (75.1)	6.158 ± 1.283	−0.333	−0.044	15.85 ± 2.645	−0.071	−0.011
Multipara	80 (24.9)	6.213 ± 1.222			15.88 ± 2.730		
**Type of delivery**							
Vaginal	85 (26.5)	6.341 ± 1.292	1.442	0.181	16.09 ± 2.869	0.959	0.117
Cesarean section	236 (73.5)	6.111 ± 1.254			15.77 ± 2.584		
**Postpartum depression**							
Yes (EPDS score ≥ 13)	301 (93.8)	6.103 ± 1.260	−5.231**	−1.009	15.74 ± 2.639	−3.064**	−0.732
No (EPDS score < 13)	20 (6.2)	7.200 ± 0.880			17.60 ± 2.437		
**EBF before discharge**							
Yes	45 (14.0)	6.933 ± 1.101	4.479**	0.752	17.42 ± 2.482	4.374**	0.715
No	276 (86.0)	6.047 ± 1.250			15.60 ± 2.606		
**Nipple depression**							
Yes	43 (13.4)	6.012 ± 1.193	−0.890	−0.149	16.12 ± 2.683	0.687	0.012
No	278 (86.6)	6.196 ± 1.278			15.82 ± 2.661		
**History of breastfeeding**							
Yes	75 (23.4)	6.227 ± 1.231	0.429	0.057	16.05 ± 2.726	0.730	0.093
No	246 (76.6)	6.155 ± 1.279			15.80 ± 2.645		
**Preterm infant’s sex**							
Male	172 (53.6)	6.227 ± 1.287	0.846	0.095	15.97 ± 2.664	0.784	0.090
Female	149 (46.4)	6.107 ± 1.243			15.73 ± 2.663		
**Preterm infant’s birth weight**							
<1,500 g	21 (6.5)	6.214 ± 1.251	0.159	0.036	16.43 ± 2.481	1.018	0.237
≥1,500 g	300 (93.5)	6.169 ± 1.269			15.82 ± 2.673		
**Time to start sucking breast milk after delivery**							
Within 2 h postpartum	11 (3.4)	5.773 ± 1.232	1.255	0.014	14.45 ± 2.382	2.415	0.018
After 2 h postpartum	83(25.9)	6.325 ± 1.170			16.17 ± 2.556		
Maternal–infant separation	227 (70.7)	6.135 ± 1.300			15.81 ± 2.698		

a Breastfeeding intention score is the total score of the Infant Feeding Intention Scale.

b Breastfeeding behavior score is the total score of the Breastfeeding Behavioral Questionnaire.

* Means *p* < 0.05, and ** means *p* < 0.01.

### Correlation analysis with PBC, ATT, SN, knowledge, breastfeeding intention, and behavior

Significant correlation coefficients (*p* < 0.01) were identified between variables of PBC, ATT, SPS, knowledge, breastfeeding intention, and behavior (Table 2), with large (*r* > 0.5) or medium effects (0.3 ≤ *r* < 0.5). Thus, all structural variables of TPB were related and included in the model testing.

**Table 2:** Correlation coefficients of the relationships between breastfeeding behavior and PBC, ATT, SPS, knowledge, and breastfeeding intention (*r*-values).

Variables	PBC^a^	ATT^b^	SPS^c^	Knowledge^d^	Intention^e^	Behavior^f^
PBC	1	--	--	--	--	--
ATT	0.498^**^	1	--	--	--	--
SPS	0.444^**^	0.437^**^	1	--	--	--
Knowledge	0.374^**^	0.441^**^	0.374^**^	1	--	--
Intention	0.562^**^	0.555^**^	0.533^**^	0.492^**^	1	--
Behavior	0.602^**^	0.506^**^	0.440^**^	0.440^**^	0.757^**^	1

a PBC score is the total score of the Breastfeeding Control Scale.

b ATT score is the total score of the Positive Breastfeeding Sentiment Attitudinal Scale.

c SPS score is the total score of the Social and Professional Support Scale.

d Knowledge score is the total score of the Breastfeeding Knowledge Questionnaire.

e Intention score is the total score of the Infant Feeding Intention Scale.

f Behavior score is the total score of the Breastfeeding Behavioral Questionnaire.

^**^Means *p* < 0.01, and --represents duplicate data.

### Model construction, examination, and adjustment

The hypothesized model was adjusted according to the modified index and the inconspicuous paths (the direct impact of other factors on breastfeeding behavior) were deleted (*p* > 0.05); the modified model was obtained with a path coefficient *p* < 0.05 (Figure 1B). The predictive constructs of intention explained 50% of its variance and the constructs of intention and PBC predicted 61% of the variance of behavior. The results of fitness indices suggest an acceptable modified model (RMSEA = 0.076, *χ*^2^/df = 2.851, GFI = 0.969, AGFI = 0.919, NFI = 0.954, RFI = 0.908, IFI = 0.969, TLI = 0.938, CFI = 0.969).

Table 3 shows the path coefficients of the modified model. Breastfeeding intention was positively influenced by PBC (*β* = 0.262, *p* < 0.01), ATT (*β* = 0.214, *p* < 0.01), SPS (*β* = 0.222, *p* < 0.01), knowledge (*β* = 0.210, *p* < 0.01), and EBF before discharge (*β* = 0.094, *p* < 0.05). However, depression negatively influenced breastfeeding intention (*β* = −0.091, *p* < 0.05), supporting *H1*.

**Table 3:** Path coefficients of the modified model.

Path hypothesis	Standardized coefficient (ß)	SE	CR	*p*-value	Results
Intention^a^ ➔ Behavior^b^	0.604	0.089	14.401	<0.01	Support
PBC^c^ ➔ Behavior^b^	0.262	0.016	6.254	<0.01	Support
PBC^c^ ➔ Intention^a^	0.269	0.008	5.564	<0.01	Support
ATT^d^ ➔ Intention^a^	0.214	0.001	4.331	<0.01	Support
SPS^e^ ➔ Intention^a^	0.222	0.001	4.738	<0.01	Support
Knowledge^f^ ➔ Intention^a^	0.210	0.019	4.587	<0.01	Support
Depression^g^ ➔ Intention^a^	−0.091	0.202	−2.296	0.022	Support
EBF before discharge ➔ Intention^a^	0.094	0.141	2.382	0.017	Support

a Intention score is the total score of the Infant Feeding Intention Scale.

b Behavior score is the total score of the Breastfeeding Behavioral Questionnaire.

c PBC score is the total score of the Breastfeeding Control Scale.

d ATT score is the total score of the Positive Breastfeeding Sentiment Attitudinal Scale.

e SPS score is the total score of the Social and Professional Support Scale.

f Knowledge score is the total score of the Breastfeeding Knowledge Questionnaire.

g Depression score is the total score of the Edinburgh Postnatal Depression Scale.

The modified model showed that only PBC (*β* = 0.262, *p* < 0.01) and intention (*β* = 0.604, *p* < 0.01) directly positively impacted breastfeeding behavior, partially supporting *H2*.

### Mediating effect analysis

Table 4 shows the standardized effects of the modified breastfeeding behavior model in total, direct, and indirect states. The indirect effect value of PBC on breastfeeding behavior was 0.162 (95% CI 0.104, 0.227; *p* < 0.01), and the direct effect was 0.262 (95% CI 0.174, 0.351; *p* < 0.01), indicating that breastfeeding intention partially mediates PBC and breastfeeding behavior; the intermediary effect accounted for 38.1% (0.162/ 0.425) of the total effect. The indirect effect of ATT on breastfeeding behavior was 0.129 (95% CI 0.066, 0.201; *p* < 0.01), and the direct effect was insignificant (*p* > 0.05), indicating that breastfeeding intention fully mediates ATT and breastfeeding behavior. The indirect effect values of SPS, knowledge, depression, and EBF before discharge on breastfeeding behavior were 0.134 (95% CI: 0.076, 0.195), 0.127 (95% CI: 0.059, 0.192), −0.055 (95% CI −0.094, −0.019), and 0.057 (95% CI: 0.014, 0.104), respectively. The *value of p* of these relationships was <0.01, and breastfeeding intention fully mediated SPS, knowledge, depression, and EBF before discharge on breastfeeding behavior via breastfeeding intention, supporting *H3*.

**Table 4:** Bootstrap results of the mediating effects of breastfeeding intention.

Path relationships	Estimate	Bias-corrected 95% CI			Percentile 95% CI		
		Lower	Upper	*P*	Lower	Upper	*P*
**Standardized indirect effect**							
PBC^a^ ➔ Intention^b^ ➔ Behavior^c^	0.162	0.104	0.227	0.001	0.112	0.215	0.001
ATT^d^ ➔ Intention^b^ ➔ Behavior^c^	0.129	0.066	0.201	0.001	0.075	0.187	0.001
SPS^e^ ➔ Intention^b^ ➔ Behavior^c^	0.134	0.076	0.195	0.001	0.085	0.183	0.001
Knowledge^f^ ➔ Intention^b^ ➔ Behavior^c^	0.127	0.059	0.192	0.001	0.069	0.183	0.001
Depression^g^ ➔ Intention^b^ ➔ Behavior^c^	−0.055	−0.094	−0.019	0.003	−0.087	−0.023	0.004
EBF before discharge ➔ Intention^b^ ➔ Behavior^c^	0.057	0.014	0.104	0.008	0.022	0.095	0.009
**Standardized direct effect**							
Intention^b^ ➔ Behavior^c^	0.604	0.530	0.676	0.001	0.544	0.665	0.001
PBC^a^ ➔ Behavior^c^	0.262	0.174	0.351	0.001	0.186	0.337	0.001
**Standardized total effect**							
Intention^b^ ➔ Behavior^c^	0.604	0.530	0.676	0.001	0.544	0.665	0.001
PBC^a^ ➔ Behavior^c^	0.425	0.343	0.502	0.001	0.356	0.490	0.001
ATT^d^ ➔ Behavior^c^	0.129	0.066	0.201	0.001	0.075	0.187	0.001
SPS^e^ ➔ Behavior^c^	0.134	0.076	0.195	0.001	0.085	0.183	0.001
Knowledge^f^ ➔ Behavior^c^	0.127	0.059	0.192	0.001	0.069	0.183	0.001
Depression^g^ ➔ Behavior^c^	−0.055	−0.094	−0.019	0.003	−0.087	−0.023	0.004
EBF before discharge ➔ Behavior^c^	0.057	0.014	0.104	0.008	0.022	0.095	0.009

a PBC score is the total score of the Breastfeeding Control Scale.

b Intention score is the total score of the Infant Feeding Intention Scale.

c Behavior score is the total score of the Breastfeeding Behavioral Questionnaire.

d ATT score is the total score of the Positive Breastfeeding Sentiment Attitudinal Scale.

e SPS score is the total score of the Social and Professional Support Scale.

f Knowledge score is the total score of the Breastfeeding Knowledge Questionnaire.

g Depression score is the total score of the Edinburgh Postnatal Depression Scale.

## Discussion

The hypotheses of this study were confirmed using model testing with acceptable fitness indices. These findings support a previous study investigating factors that contribute to breastfeeding attrition (Huang et al., 2023) and further identified that perceived breastfeeding control, breastfeeding attitude, social and professional support, knowledge, depression, and EBF before discharge are factors related to both breastfeeding intention and behavior. Prior study by our group determined that breastfeeding intention is a key factor in attrition; here, we further investigated the mechanisms of factors related to breastfeeding behavior and the mediating role of breastfeeding intention. Notably, we confirmed that breastfeeding intention is a key factor in breastfeeding behavior in preterm mothers and further identified that it fully mediates breastfeeding attitude, social and professional support, knowledge, depression, and EBF before discharge on breastfeeding behavior. Therefore, the results of the model testing further confirmed the theory of TPB, which generally suggests that the intention to perform goal-driven behaviors is critical to actually performing that behavior (Ajzen, 2011). Hence, preterm mothers’ breastfeeding attitude, social and professional support, perceived breastfeeding control, and other factors (including their knowledge, depression, and EBF before discharge) significantly influenced their breastfeeding behavior via fully or partially influencing breastfeeding intention.

These findings suggest that perceived breastfeeding control is the strongest predictor of breastfeeding intention, followed by social and professional support and breastfeeding attitude. Recent studies (Dodgson et al., 2003; Bajoulvand et al., 2019) also report that perceived breastfeeding control is the best predictor of breastfeeding intention, and the central role of perceived breastfeeding control is reflective of the realistic understanding of participants of the limitations placed on the likely success of breastfeeding efforts (Dodgson et al., 2003). Indeed, preterm mothers with enhanced perceived breastfeeding control and breastfeeding skills after discharge should be able to cope with various problems that arise in breastfeeding even after 6 weeks postpartum, which might further influence EBF intention and behavior. Early intervention to promote breastfeeding may include strategies to enhance perceived breastfeeding control, practice correct breastfeeding techniques, and solve EBF problems (Gu et al., 2016).

Subjective norm, primarily regarded as social and professional support, was also a significant factor of breastfeeding intention in the current study. Research (Bar-Yam and Darby, 1997; Arora et al., 2000; Sullivan et al., 2004) suggests that breastfeeding decisions are largely influenced by intimate partners, particularly the father of the baby. Indeed, the beliefs of partners seem to matter *more* than the mother’s own beliefs in predicting breastfeeding intention and behavior (Rempel and Remple, 2004). Regardless of the partner’s beliefs, general support during breastfeeding initiation is critical for continued success, as mothers receiving support during breastfeeding initiation and beyond promote a calm and relaxed environment, which helps them produce prolactin and oxytocin for continued production and excretion of breast milk (Lawrence and Lawrence, 2005). As preterm mothers face increased challenges with breastfeeding, they usually receive specific support from family members and hospital staff (i.e., family integrated care, FICare). In the FICare programs, preterm mothers and their partners participate in educational sessions that increase their knowledge of EBF and help them understand the importance and benefits of breastfeeding, especially for preterm infants (Ding et al., 2023). While this support is beneficial to the breastfeeding decisions and intentions of the parents (Ding et al., 2023), more parents may attempt breastfeeding if supported through bedside teaching and deep involvement by hospital staff in daily feeding (breastfeeding or bottle feeding) in the NICU (Zielińska et al., 2017). Therefore, the influence of subjective norms on breastfeeding intention needs further clarification via qualitative, observational, and interventional studies.

This study also confirmed that breastfeeding attitude is influential to breastfeeding intention. Preterm infants often face a variety of breastfeeding dilemmas, such as maternal–infant separation (Victora et al., 2016), uncoordinated sucking swallowing–respiratory function (Hair et al., 2016), and failure to create appropriate negative pressure during sucking (Mangili and Garzoli, 2017). If mothers recognize these difficulties and realize that breast milk is the optimal nutrition for their preterm infants, they may establish a positive attitude toward breastfeeding and be more inclined to opt for EBF. Notably, this study found that breastfeeding knowledge positively impacts breastfeeding intention, while a previous study suggests that the level of breastfeeding knowledge influences both intention and type of feeding of the newborn (Suárez-Cotelo et al., 2019). Indeed, the positive attitude of mothers toward breastfeeding may be associated with their perceived knowledge about the benefits of breastfeeding to the growth and development of preterm infants. Mothers who receive education regarding the health and emotional benefits of EBF often improve their attitude toward breastfeeding (Bai et al., 2010). Moreover, knowledge of breastfeeding skills may promote perceived control of mothers on pumping, storage, and transportation of breast milk. Thus, when encountering difficulties in breastfeeding preterm infants, mothers should then seek a solution using their adequate breastfeeding knowledge.

This study found that EBF before discharge positively impacts breastfeeding intention. EBF before discharge depends on lactation amount, and insufficient lactation is the primary factor leading to low breastfeeding rates of preterm infants after discharge (Fewtrell et al., 2016). Lactogenesis stage II usually occurs between the 3rd and 8th day postpartum. A stratified study on the lactation volume of preterm mothers on the 4th day postpartum found that mothers whose lactation volume was at level I (<140 mL) had a 9.5-fold probability of insufficient lactation at week 6 postpartum, compared with those at level II (140–394 mL) and level III (≥395 mL; Hill, 2005). A cross-sectional study conducted in China further reported that the rate of EBF during hospitalization positively correlated with the rate of EBF at week 6 postpartum (Quan and Yang, 2005). These findings may partially explain the results of the current study, although the rate (only 14%) of EBF before discharge was low among preterm mothers in this study. However, the low rate of EBF before discharge does suggest that nurses should strengthen breastfeeding education during hospitalization to avoid delayed initiation of lactogenesis stage II, which is the key to the success of EBF.

In this study, 93.8% of preterm mothers had an EPDS incidence score ≥ 13 during hospitalization, and their level of depressive symptoms negatively impacted their intention to breastfeed. Preterm birth is a traumatic event for preterm mothers (Gulamani et al., 2013), and these women often have higher levels of depressive symptoms than mothers of full-term infants up to 12 weeks postpartum (Vigod et al., 2010). These results are in concordance with prior studies that reported heightened levels of stress, anxiety, and depressive symptoms both during and beyond the NICU stay of premature infants (Zhang et al., 2014). Indeed, studies have confirmed that depression affects the endocrine and metabolic functions of the body, inducing a variety of hormone-level disorders. Lactation regulation depends on prolactin, oxytocin, and other neurohormones and is vulnerable to adverse emotions. At the same time, depressed mothers are prone to fatigue, and have poor initiative to actively empty the breast, resulting in a further decline in lactation, which affects breastfeeding confidence, reduces breastfeeding intention, and leads to early breastfeeding cessation (Cohen, 1988; Dennis and McQueen, 2007; Insaf et al., 2011). Therefore, the negative experiences preterm mothers endure may be associated with high levels of depressive symptoms, which continuously weaken breastfeeding intention until and through week 6 postpartum. This study also indicates that effective intervention strategies to improve breastfeeding intention and behavior for the target population (such as parent support, psychological and emotional counseling, parent training, and infant developmental support) are essential to reducing negative emotions and further promoting prolactin secretion (Cohen, 1988; Dennis and McQueen, 2007; Zhang et al., 2014). The role of breastfeeding intention also indicates that multifaceted intervention strategies to improve breastfeeding behavior should be considered for the preterm mother, including adopting individual instruction and support on breastfeeding techniques and problem-solving to improve breastfeeding knowledge and perceived breastfeeding control of mothers; implementing group education to emphasize the benefit of EBF; rectifying cognitive misconceptions of preterm mothers regarding nutrition needs for infants to enhance maternal attitude toward breastfeeding; and sharing successful breastfeeding experiences or stories from other mothers or nursing staff to enhance subjective norms (Gu et al., 2016). In addition, the breastfeeding intention–behavioral gap should not be neglected. Previous studies have shown that implementation intention is a key strategy that helps to transform behavioral intention into behavior (Latimer et al., 2006). In other words, once an intention is formed, it is necessary to adopt an action plan, which includes the specific steps of “when, where, and how” for initiating the behavior, as well as the identification of anticipated barriers and setbacks with corresponding coping plans (Martinez-Brockman et al., 2017). Therefore, it is necessary to evaluate the role of implementation intention to promote the transformation of breastfeeding intention into breastfeeding behavior in future intervention studies.

### Study limitations and future research

This study is not without its limitations. First, the surveys utilized here were self-reports that included questions regarding breastfeeding behavior at week 6 postpartum. While prior research has shown that maternal self-reporting of breastfeeding is a reliable measure (Li et al., 2005), there is an inherent risk in this measure: Social desirability bias may lead to over-reporting. Indeed, each of the participants in the current study was aware of the optimal behavior as reported by study investigators and was later asked to report their intention to practice the behavior (Wallenborn et al., 2019). Second, no significant influence of the demographic factors of the dyads was identified on breastfeeding intention and behavior. Although this finding is consistent with that of a prior study (Tengku Ismail et al., 2016), additional longitudinal studies will help elucidate the contribution of demographic factors to breastfeeding intention. Third, the contributions of the variance in breastfeeding behavior in this study were lower than those in a study of full-term mothers (79%; Bajoulvand et al., 2019), indicating that other factors related to breastfeeding behavior need to be evaluated. Finally, the participants were recruited from one hospital in Shanghai (the largest city in China), and a random sampling approach was not used in this study. Thus, sample representativeness and generalizability of the findings could be biased. A multicentered study with a more geographically diverse sample should be considered in future studies.

## Conclusion

This study preliminarily confirmed that TPB predicts breastfeeding behavior among mothers with preterm infants at week 6 postpartum. The crucial mediating role of breastfeeding intention was also identified between breastfeeding attitude, subjective norms, perceived control, knowledge, depression, and EBF before discharge with breastfeeding behavior. The findings of this study indicate that standardized questionnaires should be included in the health information system to identify preterm mothers with a high risk of EBF attrition. The tailored intervention led by nurses should focus on the breastfeeding intention and behavior of high-risk mothers with preterm infants.

## Data availability statement

The raw data supporting the conclusions of this article will be made available by the authors, without undue reservation.

## Author contributions

RH contributed to conceptualization, methodology, and original drafts. HH, XY, and LD contributed to the acquisition of data and data analysis with the assistance of YZ, YH, CC, and JS. XL and HH participated in making SEM. SZ and HJ participated in project administration, supervision and review. All authors contributed to the article and approved the submitted version.

## Funding

This study was supported by the National Natural Science Foundation of China (grant number: 72004163), China Health Human Resources Training Programme (no. RCLX2320052), and “Reservoir” Talent Development Program of Shanghai First Maternity and Infant Hospital.

## Conflict of interest

The authors declare that the research was conducted in the absence of any commercial or financial relationships that could be construed as a potential conflict of interest.

## Publisher’s note

All claims expressed in this article are solely those of the authors and do not necessarily represent those of their affiliated organizations, or those of the publisher, the editors and the reviewers. Any product that may be evaluated in this article, or claim that may be made by its manufacturer, is not guaranteed or endorsed by the publisher.

## Figures and Tables

**Table:** 

EBF	Exclusive breastfeeding
NICU	Neonatal intensive care unit
WHO	World Health Organization
ATT	Attitude
SN	Subjective norms
SPS	Social and Professional Support (Scale)
PBC	Perceived breastfeeding control
BAPT	Breastfeeding attrition predictive tool
PBS	Positive Breastfeeding Sentiment Attitudinal Scale
BFC	Breastfeeding Control Scale
EPDS	Edinburgh Postnatal Depression Scale
BKQ	Breastfeeding Knowledge Questionnaire
IFIS	Infant Feeding Intention Scale
BBQ	Breastfeeding Behavioral Questionnaire
SEM	Structural equation modeling
RMSEA	Root mean square error of approximation
GFI	Goodness-of-fit index
AGFI	Adjusted goodness-of-fit index
TLI	Tucker–Lewis coefficient
CFI	Comparative fit index
NFI	Normed fit index
RFI	Relative fit index
IFI	Incremental fit index
